# Comparative analysis of clinicopathologic characteristics and prognosis between nasal and nonnasal extranodal NK/T‐cell lymphoma

**DOI:** 10.1002/cam4.6674

**Published:** 2023-10-30

**Authors:** Ziyuan Shen, Xicheng Chen, Cai Sun, Tianyi Lu, Yuye Shi, Hao Zhang, Jingjing Ye, Ling Wang, Taigang Zhu, Yuqing Miao, Xudong Zhang, Liang Wang, Guoqi Cai, Wei Sang

**Affiliations:** ^1^ Department of Epidemiology and Biostatistics, School of Public Health Anhui Medical University Hefei Anhui China; ^2^ Department of Hematology Affiliated Hospital of Xuzhou Medical University Xuzhou Jiangsu China; ^3^ Department of Hematology The First People's Hospital of Huai'an Huai'an Jiangsu China; ^4^ Department of Hematology The Affiliated Hospital of Jining Medical University Jining Shandong China; ^5^ Department of Hematology Qilu Hospital of Shandong University Jinan Shandong China; ^6^ Department of Hematology Tai'an Central Hospital Tai'an Shandong China; ^7^ Department of Hematology The General Hospital of Wanbei Coal‐Electric Group Suzhou Anhui China; ^8^ Department of Hematology Yancheng First People's Hospital Yancheng Jiangsu China; ^9^ Department of Hematology The First Affiliated Hospital of Zhengzhou University Zhengzhou Henan China; ^10^ Department of Hematology, Beijing Tongren Hospital Capital Medical University Beijing China; ^11^ Blood Diseases Institute, Xuzhou Medical University Xuzhou Jiangsu China; ^12^ Key Laboratory of Bone Marrow Stem Cell Xuzhou Jiangsu China

**Keywords:** clinicopathologic, extranodal natural killer/T‐cell lymphoma, nasal, nonnasal

## Abstract

**Background:**

The clinicopathologic characteristics and prognosis of nasal and nonnasal extranodal natural killer T‐cell lymphoma (ENKTL) are considered to be different. However, the underlying features responsible for these differences are not well clarified especially in the era of asparaginase therapy.

**Methods:**

In total, 1007 newly diagnosed ENKTL patients from 11 medical centers were included in this study. Clinicopathologic characteristics and survival data were collected. The chi‐squared test and Kruskal–Wallis test were utilized for the comparison of different groups. Univariable and multivariable Cox proportional hazards models were used to screen prognostic factors.

**Results:**

Overall, 869 (86.3%) patients were nasal forms. Compared to patients with nasal ENKTL, nonnasal patients were at more advanced stages and had poor performance status, bone marrow involvement, elevated serum lactate dehydrogenase (LDH), and CD56‐negative status (*p* < 0.05). The 5‐year overall survival (OS) for nasal and nonnasal patients were 65.6% and 45.0%, respectively. The OS of nasal forms patients were superior to nonnasal patients, especially in Eastern Cooperative Oncology Group performance status (ECOG PS) (≥2), advanced stage, KPI (HIR/HR), IPI (HIR/HR), PINK (HR), and high EBV DNA load groups. In patients treated with pegaspargase/L‐asparaginase‐based regimens, the OS of nasal patients was better than that of nonnasal patients. After adjusting the covariates of age, stage, ECOG PS score, LDH, B symptoms, and BM involvement, results showed that the nonnasal site was associated with poor survival of ENKTL.

**Conclusions:**

The clinicopathologic characteristics and prognosis of nasal and nonnasal ENKTL patients are different. Nasal forms patients had superior OS than nonnasal patients, especially in the era of asparaginase.

## Graphical abstract

Kaplan–Meier curves for OS of nasal and nonnasal patients according to (A) Eastern Cooperative Oncology Group performance status (ECOG PS) score; (B) Ann Arbor stage; (C) CA stage; (D) KPI; (E) IPI; (F) PINK.

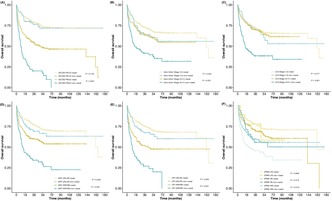

## 1 INTRODUCTION

Extranodal natural killer/T‐cell lymphoma (ENKTL) is an aggressive and heterogeneous malignancy, which is substantially more prevalent in Asian and Latin American populations.^1^, ^2^ Although the precise mechanism is still unclear, Epstein–Barr virus (EBV) infection seems to play a key role in the pathogenesis of ENKTL.^3^, ^4^, ^5^, ^6^ Most cases involve the nasal cavity and sinuses (nasal), while a few cases primarily occur in other extranodal areas such as the skin, intestines, testicles, etc. without the involvement of the nasal cavity and sinuses (nonnasal).^7^, ^8^, ^9^, ^10^, ^11^, ^12^, ^13^ Previous studies have proved that nonnasal patients had more adverse clinical features and poorer survival than nasal forms,^14^, ^15^, ^16^ but little is known about the disparities of clinicopathologic characteristics and immunohistochemical features between them. The heterogeneity in pathology, complexity of molecular pathogenesis, and genetic abnormalities pose great challenges for individualized treatment strategies.

In a multi‐omics study, the authors demonstrated that genomic alteration‐based molecular subtypes were associated with clinical outcomes and MYC, histone acetylation, and PD‐L1/2 were potential therapeutic targets of ENKTL.^17^ While treatments vary based on the patient's status, pegaspargase/L‐asparaginase‐based regimens are currently first‐line options for ENKTL patients.^18^, ^19^, ^20^, ^21^, ^22^ However, there are still newly diagnosed patients experiencing disease progression, and the prognosis in patients with relapsed or refractory ENKTL remains unsatisfactory.^23^ More effective treatment approaches deserve further exploration.^24^, ^25^ The encouraging results on anti‐PD‐1 antibodies have started to challenge the current treatment mode of ENKTL and have provided a theoretical basis for first‐line treatment of patients with advanced ENKTL.^26^, ^27^, ^28^, ^29^ Kim et al. revealed that higher international prognosis index (IPI) score and the presence of complications were adverse prognostic factors of primary gastrointestinal T‐cell lymphomas.^30^, ^31^ For nasal patients, the prognostic effect of stage, Eastern Cooperative Oncology Group performance status (ECOG PS) score, and hemoglobin level were also confirmed.^32^, ^33^ Exploring differences in clinicopathologic characteristics and immunohistochemical features between nasal and nonnasal patients and identifying adverse prognostic factors could provide options for individualized treatment.

In this study, we retrospectively recruited patients with newly diagnosed ENKTL from the Huaihai Lymphoma Working Group and evaluated the differences between nasal and nonnasal sites.

## 2 METHODS

### 2.1 Patients

A total of 1007 patients (age 12–86 years) between 2009 and 2021 from 11 medical centers in Huaihai Lymphoma Working Group (HHLWG) were enrolled. Diagnosis of ENKTL was based on criteria of World Health Organization classification. Clinical doctors from each participating hospital reviewed the conclusions of positron emission tomography combined with computed tomography (PET/CT) and pathology to determine disease staging, assess pathological results, and exclude patients with occult tumors. Ethics approval was obtained from independent Ethics Committees of each participating center in HHLWG and this study was conducted in accordance with the Declaration of Helsinki.

### 2.2 Covariates

The following variables were collected: age, gender, ECOG PS, the presence of B symptoms, bone marrow involvement (BM involvement), Ki67, EBV DNA load, and the expression of CD56, serum lactate dehydrogenase (LDH), creatinine (Cr), total cholesterol (TC), triglyceride (TG), high‐density lipoprotein (HDL), low‐density lipoprotein (LDL), albumin (ALB), globulin (GLB), white blood cell count (WBC), lymphocyte count (LYC), monocyte (MONO), hemoglobin (HGB), fibrinogen (FIB), and β2‐microglobulin (β2‐MG). All patients were staged according to the Ann Arbor Staging system and the Chinese Southwest Oncology Group and Asia Lymphoma Study Group ENKTL (CA) system.^34^, ^35^

### 2.3 Follow‐up and endpoints

Follow‐up was conducted by consulting inpatient medical records and making phone calls. The follow‐up ended in October 2022. Overall survival (OS) was calculated as the interval between the time of diagnosis and the date of death or the last follow‐up. The survival status of all patients was confirmed with death records or a telephone call to next of kin (if patient died during the follow‐up) or to the patients themselves.

### 2.4 Statistical analysis

Baseline characteristics were presented as mean (standard deviation, SD) and *n* (%). Analysis of variance, chi‐squared test and Kruskal–Wallis test were utilized for the comparison of different groups. Ki67 was transformed into categorical variable by maximally selected rank statistics (MaxStat) analysis.

The collinearity between covariates was assessed by variance inflation factor (VIF) statistic.^36^ The Cox proportional hazard model was used to explore the univariable and multivariable associations between prognostic factors and OS. All variables with *p* < 0.05 in the univariable analysis were kept in the multivariable analysis. Kaplan–Meier analysis was used to estimate the probability of OS, and survival curves were compared using the log‐rank test.

All statistical analyses were performed by R software (version 4.2.0; http://www.Rproject.org). Statistical significance was set at a *p* value of <0.05 (two‐tailed).

## 3 RESULTS

### 3.1 Patient characteristics

A total of 1007 ENKTL patients (68.5% male, mean age 46.1 ± 14.9 years [range 12.0–86.0]) were enrolled in this study, of whom 869 cases were nasal forms (ratio: 6:1). The baseline characteristics are shown in Table 1. The main between‐group differences were BM involvement, ECOG PS score, CD56, Ann Arbor stage, CA stage, KPI, IPI, PINK, LDH, ALB, LDL, GLB, HGB, and FIB. Nonnasal group patients had more adverse clinical features such as high ECOG PS score, advanced stage, and high proportion of BM involvement. The involvements of nonnasal sites were more observed in this study, including gastrointestinal tract (21.7%), pharyngeal tonsil (20.3%), and skin (12.3%).

**TABLE 1:** Baseline characteristics of nasal and nonnasal extranodal natural killer T‐cell lymphoma (ENKTL) patients.

	Nasal (*n* = 869)	Nonnasal (*n* = 138)	*p*
Age mean (SD)	46.39 (14.81)	44.67 (15.53)	0.209
Gender (%)			
Male	596 (68.6)	94 (68.1)	0.991
Female	273 (31.4)	44 (31.9)	
B symptoms (%)			
Absence	429 (49.4)	62 (44.9)	0.380
Presence	440 (50.6)	76 (55.1)	
BM involvement (%)			
Absence	846 (97.4)	123 (89.1)	<0.001
Presence	23 (2.6)	15 (10.9)	
ECOG PS (%)			
<2	615 (70.8)	75 (54.3)	<0.001
≥2	254 (29.2)	63 (45.7)	
Ki67 mean (SD)	0.61 (0.19)	0.62 (0.20)	0.783
CD56 (%)			
Negative	60 (6.9)	22 (15.9)	0.001
Positive	809 (93.1)	116 (84.1)	
EBV‐DNA (%)	364 (41.9)	46 (33.3)	0.071
Negative	505 (58.1)	92 (66.7)	
Positive			
Ann Arbor stage (%)	752 (86.5)	55 (39.9)	<0.001
I/II	117 (13.5)	83 (60.1)	
III/IV			
CA stage (%)			
I/II	681 (78.4)	52 (37.7)	<0.001
III/IV	188 (21.6)	86 (62.3)	
KPI (%)			
LR	268 (30.8)	21 (15.2)	<0.001
LIR	337 (38.8)	43 (31.2)	
HIR	175 (20.1)	36 (26.1)	
HR	89 (10.2)	38 (27.5)	
IPI (%)			
LR	572 (65.8)	54 (39.1)	<0.001
LIR	160 (18.4)	25 (18.1)	
HIR	81 (9.3)	36 (26.1)	
HR	56 (6.4)	23 (16.7)	
PINK (%)			
LR	438 (50.4)	9 (6.5)	<0.001
IR	244 (28.1)	35 (25.4)	
HR	187 (21.5)	94 (68.1)	
LDH mean (SD)	249.87 (364.57)	372.97 (609.91)	0.001
Cr mean (SD)	71.32 (32.75)	66.68 (27.62)	0.114
TC mean (SD)	4.14 (1.68)	3.86 (1.17)	0.065
TG mean (SD)	1.46 (0.80)	1.59 (0.79)	0.083
LDL mean (SD)	2.37 (0.91)	1.94 (1.00)	<0.001
HDL mean (SD)	1.13 (0.55)	1.05 (0.55)	0.108
ALB mean (SD)	37.91 (6.43)	35.53 (7.30)	<0.001
GLB mean (SD)	27.68 (5.24)	26.22 (5.24)	0.003
WBC mean (SD)	5.71 (2.43)	5.48 (2.37)	0.287
LYC mean (SD)	1.40 (0.82)	1.39 (1.12)	0.922
HGB mean (SD)	129.04 (27.08)	116.27 (20.76)	<0.001
β2‐MG mean (SD)	2.87 (1.48)	3.12 (1.58)	0.069
FIB mean (SD)	3.03 (1.25)	2.75 (1.49)	0.015

Abbreviations: ALB, albumin; BM involvement, bone marrow involvement; CA stage, the Chinese Southwest Oncology Group and Asia Lymphoma Study Group ENKTL system; FIB, fibrinogen; GLB, globulin; HGB, hemoglobin; IPI, International Prognostic Index; KPI, Korean Prognostic Index; LDL, low‐density lipoprotein; PINK, Prognostic index of natural killer lymphoma LDH, serum lactate dehydrogenase; TC, total cholesterol; β2‐MG, β2‐microglobulin.

The 5‐year OS for nasal and nonnasal patients were 65.6% and 45.0%, respectively. The median follow‐up was 61.6 (95% CI:58.1–65.0) months for nasal patients and 53.6 (95% CI:47.4–59.8) months for nonnasal patients.

### 3.2 Assessment of clinical features

**FIGURE 1:**
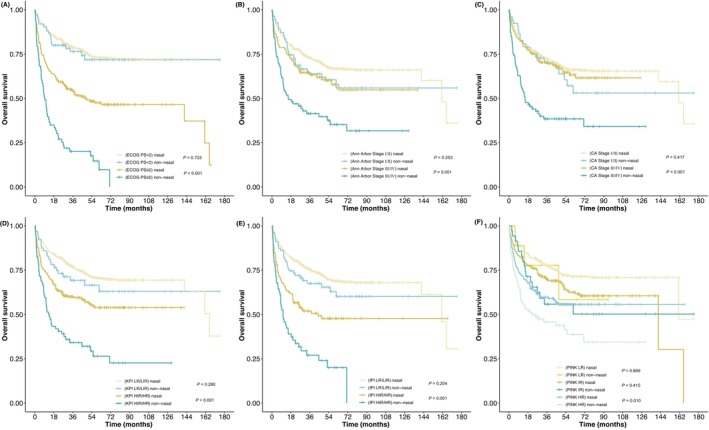
Kaplan–Meier curves for overall survival (OS) of nasal and nonnasal patients according to (A) Eastern Cooperative Oncology Group performance status (ECOG PS) score; (B) Ann Arbor stage; (C) CA stage; (D) KPI; (E) IPI; (F) PINK.

Compared with nonnasal patients, nasal patients had better OS in ECOG PS (≥2), advanced Ann Arbor stage, advanced CA Stage, KPI (HIR/HR), IPI (HIR/HR), and PINK (HR) groups (Figure 1). In nasal patients, those with higher ECOG PS score and advanced Ann Arbor stage disease had poorer OS (*p* < 0.001, *p* = 0.001). Furthermore, nonnasal patients in advanced CA stage also had poor OS (*p* < 0.001).

### 3.3 Disparity analysis of pathologic characteristics

The optimal cut‐off point for Ki67 was 0.4 by MaxStat analysis (*χ* ^2^ = 3.094, *p* = 0.002).

**FIGURE 2:**
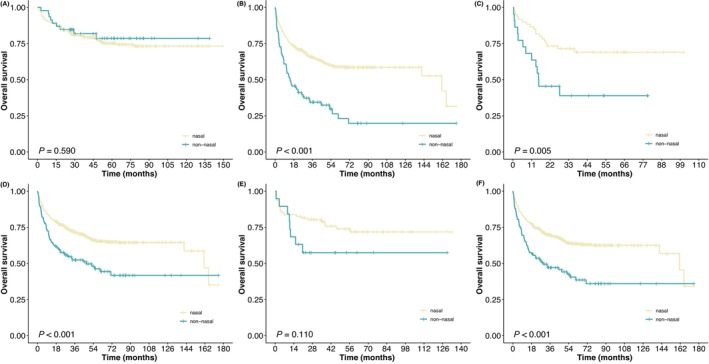
Kaplan–Meier curves for overall survival (OS) of nasal and nonnasal patients according to (A) low EBV DNA load; (B) high EBV DNA load; (C) CD56‐negative; (D) CD56‐positive; (E) Ki67 ≤ 0.4; (F) Ki67 > 0.4.

Compared with nonnasal patients, nasal patients had statistically significant superiority of OS in high EBV DNA load, CD56, and high Ki67 level groups (Figure 2). We noticed that in advanced stage (both Ann Arbor and CA stage) of nasal group, CD56‐positive patients had significantly superior OS to CD56‐negative patients (*p* = 0.044, *p* = 0.029). In nonnasal group, CD56‐positive patients had significantly superior OS in early CA stage and low ECOG PS score (*p* = 0.001, *p* < 0.001).

### 3.4 Treatment regimens

**FIGURE 3:**
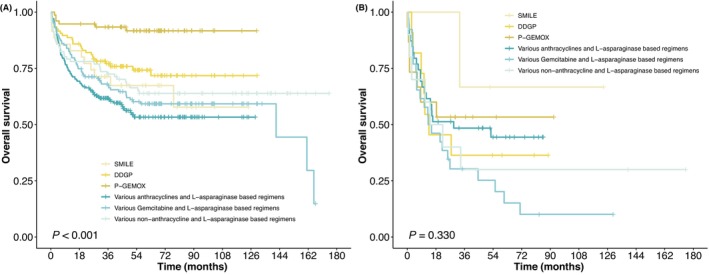
Kaplan–Meier curves for overall survival (OS) of different regimens in (A) nasal group; (B) nonnasal group.

The initial treatment regimens were as follows: 75.4% patients received chemotherapy with pegaspargase/L‐asparaginase‐based regimens, of which 32.1% patients received anthracyclines and L‐asparaginase‐based regimens, followed by gemcitabine and L‐asparaginase‐based regimens (22.2%). About 3.7% patients underwent SMILE regimens. In nasal group, 106 patients received DDGP regimens, and 76 patients received P‐GEMOX regimens. In nonnasal group, 10.5% patients received DDGP regimens, and only 2.8% patients underwent SMILE regimens. The survival of patients who received P‐GEMOX regimen was superior to those who underwent SMILE regimen in nasal group (*p* < 0.001, Figure 3A). DDGP chemotherapy resulted in significant improvement in OS and better tolerability compared with anthracyclines and L‐asparaginase‐based regimens chemotherapy (*p* = 0.002). No significant difference was found in the treatment modalities of nonnasal group (Figure 3B).

Nasal forms ENKTL patients treated with pegaspargase/L‐asparaginase‐based regimens had better survival than that of nonnasal patients (Figure S1A). While, no significant difference was found between nasal and nonnasal patients in without pegaspargase/L‐asparaginase‐based regimens group (Figure S1B). Nasal forms ENKTL patients treated with DDGP, P‐GEMOX, gemcitabine‐ and L‐asparaginase‐based, and non‐anthracyclines and L‐asparaginase‐based regimens also had better survival than that of nonnasal patients (Figure S1D,E,G,H).

### 3.5 Survival analyses

The results showed that there was no interrelationships among independent variables (VIF < 5). Table 2 summarizes the results of univariable analysis of nasal and nonnasal patients. ECOG PS, BM involvement, EBV DNA‐positive, and β2‐MG appeared to be stronger predictors in both groups (*p* < 0.05).

**TABLE 2:** Univariable analyses of prognostic factors for overall survival in nasal and nonnasal groups.

Variables	Nasal			Nonnasal		
	HR	95% CI	*p*	HR	95% CI	*p*
Age	1.008	1.000–1.016	0.057	1.004	0.988–1.019	0.649
Gender (female)	0.800	0.617–1.037	0.092	0.804	0.484–1.335	0.399
ECOG PS (≥2)	2.579	2.038–3.263	<0.001	6.315	3.697–10.786	<0.001
Ann Arbor stage (III/IV)	1.443	1.051–1.980	0.023	2.127	1.280–3.534	0.004
CA stage (III/IV)	1.117	0.843–1.479	0.441	2.231	1.331–3.739	0.002
B symptoms (presence)	1.841	1.446–2.344	<0.001	1.821	1.126–2.943	0.014
BM involvement (presence)	2.626	1.504–4.587	0.001	3.085	1.673–5.687	<0.001
EBV DNA (positive)	1.859	1.438–2.404	<0.001	5.874	2.906–11.873	<0.001
CD56 (positive)	1.057	0.656–1.705	0.819	0.750	0.411–1.367	0.347
LDH	1.001	1.000–1.001	<0.001	1.001	1.000–1.001	<0.001
TC	0.805	0.715–0.905	<0.001	0.836	0.667–1.047	0.119
TG	1.265	1.118–1.431	<0.001	1.169	0.918–1.488	0.205
LDL	0.777	0.678–0.889	<0.001	0.790	0.617–1.012	0.062
HDL	0.499	0.370–0.673	<0.001	0.400	0.234–0.685	0.001
ALB	0.958	0.941–0.975	<0.001	0.957	0.930–0.984	0.002
HGB	0.982	0.976–0.988	<0.001	0.982	0.971–0.994	0.002
β2‐MG	1.209	1.141–1.282	<0.001	1.237	1.089–1.404	0.001
Ki67 (>40%)	1.586	1.131–2.225	0.008	1.700	0.895–3.228	0.105
Cr	1.002	0.999–1.006	0.192	1.005	0.998–1.013	0.134
FIB	0.968	0.877–1.069	0.526	0.830	0.703–0.979	0.027
GLB	0.989	0.966–1.012	0.332	0.960	0.920–1.002	0.063
WBC	1.006	0.957–1.057	0.814	1.021	0.918–1.134	0.704
LYC	0.888	0.754–1.046	0.155	0.618	0.431–0.887	0.009

Abbreviations: ALB, albumin; BM involvement, bone marrow involvement; FIB, fibrinogen; GLB, globulin; HDL, high‐density lipoprotein; HGB, hemoglobin; LDH, serum lactate dehydrogenase; LDL, low‐density lipoprotein; TC, total cholesterol; TG, triglyceride; β2‐MG, β2‐microglobulin.

Following the model iterations in multivariable analysis, seven independent prognostic factors was associated with nasal ENKTL patients. CA advanced stage, high ECOG PS score and EBV DNA‐positive status were adverse factors for nonnasal ENKTL patients (Table 3).

**TABLE 3:** Multivariable analyses of prognostic factors of nasal and nonnasal patients.

	Variables	HR	95% CI	*p*
Nasal	ECOG PS (≥2)	2.346	1.846–2.981	<0.001
	LDH	1.001	1–1.001	<0.001
	TC	0.801	0.71–0.904	<0.001
	TG	1.196	1.051–1.36	0.007
	HGB	0.99	0.984–0.996	0.001
	Ki67 (>40%)	1.483	1.05–2.095	0.025
	EBV DNA (positive)	1.51	1.157–1.972	0.002
Nonnasal	CA stage (III/IV)	1.973	1.132–3.439	0.017
	ECOG PS (≥2)	4.17	2.38–7.304	<0.001
	EBV DNA (positive)	3.214	1.525–6.773	0.002

Abbreviations: HGB, hemoglobin; LDH, serum lactate dehydrogenase; TC, total cholesterol; TG, triglyceride.

In the whole cohort, after adjusting the covariates of age, stage, ECOG PS score, LDH B symptoms, and BM involvement, the results demonstrated that nonnasal was associated with poor prognosis of ENKTL (HR = 1.392, 95% CI:1.034–1.873, *p* = 0.029).

## 4 DISCUSSION

In this retrospective multicenter study, we found that the clinical characteristics and pathological features between nasal and nonnasal patients were largely different. In general, nonnasal forms was an adverse factor for the prognosis of ENKTL, and nonnasal patients were with poorer survival than that of nasal patients especially in the era of asparaginase.

ENKTL is an invasive and heterogeneous lymphoma. Based on different subtypes, the clinicopathologic characteristics are significantly different. As two main subtypes of ENKTL, several researches have separately analyzed the clinical characteristics and prognosis of nasal and nonnasal patients.^37^, ^38^ However, there are few parallel and comparative studies to explore the disparity of clinicopathologic features between nasal and nonnasal.^39^, ^40^ In this study, we found that the 5‐year OS of nonnasal was poorer than nasal forms, and nonnasal ENKTL patients showed worse clinical characteristics, which were consistent with previous research results.^14^ No significant difference in the 5‐year OS rate was found among nonnasal that involved gastrointestinal tract, pharyngeal tonsil, and skin. Nonnasal patients were at more advanced stage and had higher ECOG PS score. In addition, nonnasal patients had worse prognosis, as indicated by IPI, KPI, and PINK.

EBV transforms the host cells from a resting state to a malignant activated state, and facilitates the oncogenesis of lymphomas.^41^ The importance of EBV to ENKTL cannot be ignored. We proved that in both nasal and nonnasal groups, patients with high EBV DNA had poorer survival. Ki67 is a nuclear and nucleolar protein antigen and the expression evaluated through immunohistochemistry is useful as one of the most reliable indicators of the proliferative status of cancer cells.^42^, ^43^ In our study, we explored the prognostic value of Ki67 and especially calculated the cut‐off point of Ki67 by Maxstat analysis. Our data demonstrated a notable finding that high Ki67 expression (>40%) was an adverse marker for nasal forms ENKTL. Previous studies reported that CD56‐negative ENKTL should be considered as a distinct subtype, and our previous study proved that CD56‐positive patients had superior OS than CD56‐negative.^44^ In this current study, we confirmed that CD56‐positive had significantly superior OS to CD56‐negative especially in nasal forms ENKTL patients with advanced stage.

L‐asparaginase‐containing treatment regimens has contributed to improving the prognosis of ENKTL patients.^45^ In this study, no significant difference was found in nasal and nonnasal patients treated without asparaginase‐based regimens. However, nasal patients treated with asparaginase‐based regimens had superior OS to nonnasal patients, which could be attributed to high proportion of advanced stage in nonnasal patients. This suggests that asparaginase‐based regimens could potentially overcome adverse prognostic factors in patients with nasal forms. Of note, no statistical difference was found in nasal and nonnasal patients treated with SMILE regimens. SMILE chemotherapy often accompanies with high hematological toxicity and is not recommended for patients with a low lymphocyte count.^46^, ^47^ Multivariable results showed that advanced stage was an adverse factor for nonnasal patients, so more promising and novel treatment approaches are needed for patients with advanced stage disease in this modern ENKTL treatment era.

The strengths of this study lie in its large sample size, long‐term follow‐up, and inclusion of multicenter data from ENKTL patients. Additionally, our study offers valuable insights into the distinctions in clinicopathologic characteristics and prognoses between nasal and nonnasal patients. Limitations of the current study are worth noting. First, we did not collect the immune markers and data of genetic measurements of all patients. Second, due to the heterogeneity of EBV DNA measured in multicenter, it was difficult to determine the optimal cut‐off point and hard to get dynamic data detection.

In conclusion, based on this long‐term, multicenter study, we found that the clinicopathologic characteristics and survival between nasal and nonnasal ENKTL patients are different. Nasal forms patients had superior OS than nonnasal patients, especially in the era of asparaginase. For advanced nonnasal patients, more novel and promising treatment regimens are needed.

## AUTHOR CONTRIBUTIONS

**Ziyuan Shen:** Data curation (equal); formal analysis (equal); writing – original draft (equal). **Xicheng Chen:** Data curation (equal). **Cai Sun:** Writing – original draft (equal). **Tianyi Lu:** Data curation (equal). **Yuye Shi:** Data curation (equal). **Hao Zhang:** Data curation (equal). **Jingjing Ye:** Data curation (equal). **Ling Wang:** Data curation (equal). **Taigang Zhu:** Data curation (equal). **Yuqing Miao:** Formal analysis (equal). **Xudong Zhang:** Data curation (equal). **Liang Wang:** Data curation (equal). **Guoqi Cai:** Conceptualization (equal); writing – review and editing (equal). **Wei Sang:** Conceptualization (equal); funding acquisition (equal); investigation (equal); writing – original draft (equal); writing – review and editing (equal).

## FUNDING INFORMATION

This study was funded by the Natural Science Foundation of Jiangsu Province, Grant/Award Number BK20171181; Jiangsu Key Research and Development Project of Social Development, Grant/Award Number BE2019638; and Young Medical Talents of Jiangsu Science and Education Health Project, Grant/Award Number QNRC2016791.

## CONFLICT OF INTEREST STATEMENT

The authors declare that they have no conflict of interest.

## ETHICS STATEMENT

This study was approved by the independent Ethics Committees of each participating center in HHLWG and conformed to the provisions of the Declaration of Helsinki.

## Supporting information


Figure S1.

## Data Availability

The datasets generated and/or analyzed during the current study are available from the corresponding author on reasonable request.

## References

[1] Lin GW , Xu C , Chen K , et al. Genetic risk of extranodal natural killer T‐cell lymphoma: a genome‐wide association study in multiple populations. Lancet Oncol. 2020;21(2):306‐316. 31879220 10.1016/S1470-2045(19)30799-5

[2] Jiang L , Gu ZH , Yan ZX , et al. Exome sequencing identifies somatic mutations of DDX3X in natural killer/T‐cell lymphoma. Nat Genet. 2015;47(9):1061‐1066. 26192917 10.1038/ng.3358

[3] Vockerodt M , Yap LF , Shannon‐Lowe C , et al. The Epstein‐Barr virus and the pathogenesis of lymphoma. J Pathol. 2015;235(2):312‐322. 25294567 10.1002/path.4459

[4] Mustafa N , Nee AHF , Chooi JY , et al. Determinants of response to daratumumab in Epstein‐Barr virus‐positive natural killer and T‐cell lymphoma. J Immunother Cancer. 2021;9(7):e002123. 34215687 10.1136/jitc-2020-002123 PMC8256838

[5] Tse E , Kwong YL . How I treat NK/T‐cell lymphomas. Blood. 2013;121(25):4997‐5005. 23652805 10.1182/blood-2013-01-453233

[6] Shen Z , Hu L , Yao M , et al. Disparity analysis and prognostic value of pretreatment whole blood Epstein‐Barr virus DNA load and Epstein‐Barr encoding region status in lymphomas: a retrospective multicenter study in Huaihai Lymphoma Working Group. Int J Cancer. 2022;150(2):327‐334. 34520566 10.1002/ijc.33802

[7] Li YX , Fang H , Liu QF , et al. Clinical features and treatment outcome of nasal‐type NK/T‐cell lymphoma of Waldeyer ring. Blood. 2008;112(8):3057‐3064. 18676879 10.1182/blood-2008-05-160176

[8] Maciejka‐Kemblowska L , Chaber R , Wrobel G , et al. Clinical features and treatment outcomes of peripheral T‐cell lymphoma in children. A current data report from Polish Pediatric Leukemia/Lymphoma Study Group (PPLLSG). Adv Med Sci. 2016;61(2):311‐316. 27254421 10.1016/j.advms.2016.03.002

[9] Suzuki R . Pathogenesis and treatment of extranodal natural killer/T‐cell lymphoma. Semin Hematol. 2014;51(1):42‐51. 24468315 10.1053/j.seminhematol.2013.11.007

[10] Matsuda M , Iwanaga T , Hashimoto S , Uesugi T , Itagaki N . Primary Epstein‐Barr virus‐negative nasal‐type natural killer/T cell lymphoma of the testis. Leuk Res. 2009;33(8):e119‐e120. 19285723 10.1016/j.leukres.2009.02.019

[11] Chang SH , Kao JH , Liang JD , Wu SJ . Successful treatment of nasal‐type extra‐nodal natural killer/T cell lymphoma with simultaneous involvement of the thyroid, liver, and pancreas. Ann Hematol. 2019;98(9):2243‐2246. 31280335 10.1007/s00277-019-03752-5

[12] Ooi G , Chim C , Liang R , Tsang K , Kwong Y . Nasal T‐cell/natural killer cell lymphoma: CT and MR imaging features of a new clinicopathologic entity. Am J Roentgenol. 2000;174(4):1141‐1145. 10749267 10.2214/ajr.174.4.1741141

[13] Alaggio R , Amador C , Anagnostopoulos I , et al. The 5th edition of the World Health Organization classification of haematolymphoid tumours: lymphoid neoplasms. Leukemia. 2022;36(7):1720‐1748. 35732829 10.1038/s41375-022-01620-2 PMC9214472

[14] Au WY , Weisenburger DD , Intragumtornchai T , et al. Clinical differences between nasal and extranasal natural killer/T‐cell lymphoma: a study of 136 cases from the international peripheral T‐cell lymphoma project. Blood. 2009;113(17):3931‐3937. 19029440 10.1182/blood-2008-10-185256

[15] Vose J , Armitage J , Weisenburger D . International peripheral T‐cell and natural killer/T‐cell lymphoma study: pathology findings and clinical outcomes. J Clin Oncol. 2008;26(25):4124‐4130. 18626005 10.1200/JCO.2008.16.4558

[16] Yamaguchi M , Suzuki R , Miyazaki K , et al. Improved prognosis of extranodal NK/T cell lymphoma, nasal type of nasal origin but not extranasal origin. Ann Hematol. 2019;98(7):1647‐1655. 31001658 10.1007/s00277-019-03689-9

[17] Xiong J , Cui BW , Wang N , et al. Genomic and transcriptomic characterization of natural killer T cell lymphoma. Cancer Cell. 2020;37(3):403‐419.e6. 32183952 10.1016/j.ccell.2020.02.005

[18] Li X , Cui Y , Sun Z , et al. DDGP versus SMILE in newly diagnosed advanced natural killer/T‐cell lymphoma: a randomized controlled, multicenter, open‐label study in China. Clin Cancer Res. 2016;22(21):5223‐5228. 27060152 10.1158/1078-0432.CCR-16-0153

[19] van Doesum JA , Niezink AGH , Huls GA , Beijert M , Diepstra A , van Meerten T . Extranodal natural killer/T‐cell lymphoma, nasal type: diagnosis and treatment. Hemasphere. 2021;5(2):e523. 33458595 10.1097/HS9.0000000000000523 PMC7806244

[20] Wang JJ , Dong M , He XH , et al. GDP (gemcitabine, dexamethasone, and cisplatin) is highly effective and well‐tolerated for newly diagnosed stage IV and relapsed/refractory extranodal natural killer/T‐cell lymphoma, nasal type. Medicine (Baltimore). 2016;95(6):e2787. 26871836 10.1097/MD.0000000000002787 PMC4753932

[21] Ji J , Li L , She NN , Liu XH , Long Y , Zhang XB . Effectiveness of P‐Gemox chemotherapy combined with radiotherapy in newly diagnosed, stage E to E, extranodal nasal type natural killer/T‐cell lymphoma. Lin Chung Er Bi Yan Hou Tou Jing Wai Ke Za Zhi. 2019;33(2):132‐137. 10.13201/j.issn.1001-1781.2019.02.010 30808138

[22] Kwong YL , Kim WS , Lim ST , et al. SMILE for natural killer/T‐cell lymphoma: analysis of safety and efficacy from the Asia Lymphoma Study Group. Blood. 2012;120(15):2973‐2980. 22919026 10.1182/blood-2012-05-431460

[23] Lim SH , Hong JY , Lim ST , et al. Beyond first‐line non‐anthracycline‐based chemotherapy for extranodal NK/T‐cell lymphoma: clinical outcome and current perspectives on salvage therapy for patients after first relapse and progression of disease. Ann Oncol. 2017;28(9):2199‐2205. 28911074 10.1093/annonc/mdx316

[24] Rong QX , Wang F , Guo ZX , et al. GM‐CSF mediates immune evasion via upregulation of PD‐L1 expression in extranodal natural killer/T cell lymphoma. Mol Cancer. 2021;20(1):80. 34051805 10.1186/s12943-021-01374-y PMC8164269

[25] Chen M , Jiang Y , Cai X , Lu X , Chao H . Combination of gemcitabine and thymosin alpha 1 exhibit a better anti‐tumor effect on nasal natural killer/T‐cell lymphoma. Int Immunopharmacol. 2021;98:107829. 34119916 10.1016/j.intimp.2021.107829

[26] Cai J , Liu P , Huang H , et al. Combination of anti‐PD‐1 antibody with P‐GEMOX as a potentially effective immunochemotherapy for advanced natural killer/T cell lymphoma. Signal Transduct Target Ther. 2020;5(1):289. 33376237 10.1038/s41392-020-00331-3 PMC7772337

[27] Kwong YL , Chan TSY , Tan D , et al. PD1 blockade with pembrolizumab is highly effective in relapsed or refractory NK/T‐cell lymphoma failing l‐asparaginase. Blood. 2017;129(17):2437‐2442. 28188133 10.1182/blood-2016-12-756841

[28] Li J , Tao R , Fan L , et al. Sintilimab for relapsed/refractory (r/r) extranodal NK/T cell lymphoma (ENKTL): extended follow‐up on the multicenter, single‐arm phase II trail (ORIENT‐4). Signal Transduct Target Ther. 2020;3:8050.

[29] Liu P , Pan X , Chen C , et al. Nivolumab treatment of relapsed/refractory Epstein‐Barr virus‐associated hemophagocytic lymphohistiocytosis in adults. Blood. 2020;135(11):826‐833. 31914172 10.1182/blood.2019003886

[30] Kim DH , Lee D , Kim JW , et al. Endoscopic and clinical analysis of primary T‐cell lymphoma of the gastrointestinal tract according to pathological subtype. J Gastroenterol Hepatol. 2014;29(5):934‐943. 24325295 10.1111/jgh.12471

[31] International Non‐Hodgkin's Lymphoma Prognostic Factors Project . A predictive model for aggressive non‐Hodgkin's lymphoma. N Engl J Med. 1993;329(14):987‐994. 8141877 10.1056/NEJM199309303291402

[32] Tan KM , Chia B , Lim JQ , et al. A clinicohaematological prognostic model for nasal‐type natural killer/T‐cell lymphoma: A multicenter study. Sci Rep. 2019;9(1):14961. 31628410 10.1038/s41598-019-51522-0 PMC6802199

[33] Wang L , Xia ZJ , Lu Y , et al. A modified international prognostic index including pretreatment hemoglobin level for early stage extranodal natural killer/T cell lymphoma. Leuk Lymphoma. 2015;56(11):3038‐3044. 25747971 10.3109/10428194.2015.1026817

[34] Cheson BD , Fisher RI , Barrington SF , et al. Recommendations for initial evaluation, staging, and response assessment of Hodgkin and non‐Hodgkin lymphoma: the Lugano classification. J Clin Oncol. 2014;32(27):3059‐3068. 25113753 10.1200/JCO.2013.54.8800 PMC4979083

[35] Hong H , Li Y , Lim ST , et al. A proposal for a new staging system for extranodal natural killer T‐cell lymphoma: a multicenter study from China and Asia lymphoma study group. Leukemia. 2020;34(8):2243‐2248. 32066865 10.1038/s41375-020-0740-1 PMC7387308

[36] Marcoulides KM , Raykov T . Evaluation of variance inflation factors in regression models using latent variable modeling methods. Educ Psychol Meas. 2019;79(5):874‐882. 31488917 10.1177/0013164418817803 PMC6713981

[37] Pagano L , Gallamini A , Trapè G , et al. NK/T‐cell lymphomas ‘nasal type’: an Italian multicentric retrospective survey. Ann Oncol. 2006;17(5):794‐800. 16497823 10.1093/annonc/mdl015

[38] Lee J , Suh C , Park YH , et al. Extranodal natural killer T‐cell lymphoma, nasal‐type: a prognostic model from a retrospective multicenter study. J Clin Oncol. 2006;24(4):612‐618. 16380410 10.1200/JCO.2005.04.1384

[39] Chan JK , Sin VC , Wong KF , et al. Nonnasal lymphoma expressing the natural killer cell marker CD56: a clinicopathologic study of 49 cases of an uncommon aggressive neoplasm. Blood. 1997;89(12):4501‐4513. 9192774

[40] Lim ST , Hee SW , Quek R , et al. Comparative analysis of extra‐nodal NK/T‐cell lymphoma and peripheral T‐cell lymphoma: significant differences in clinical characteristics and prognosis. Eur J Haematol. 2008;80(1):55‐60. 18028433 10.1111/j.1600-0609.2007.00978.x

[41] Cai Q , Cai J , Fang Y , Young KH . Epstein‐Barr virus‐positive natural killer/T‐cell lymphoma. Front Oncol. 2019;9:386. 31139570 10.3389/fonc.2019.00386 PMC6527808

[42] Endl E , Gerdes J . The Ki‐67 protein: fascinating forms and an unknown function. Exp Cell Res. 2000;257(2):231‐237. 10837136 10.1006/excr.2000.4888

[43] Jiang L , Li P , Wang H , et al. Prognostic significance of Ki‐67 antigen expression in extranodal natural killer/T‐cell lymphoma, nasal type. Med Oncol. 2014;31(10):218. 25204411 10.1007/s12032-014-0218-y

[44] Wang L , Wang Z , Xia ZJ , Lu Y , Huang HQ , Zhang YJ . CD56‐negative extranodal NK/T cell lymphoma should be regarded as a distinct subtype with poor prognosis. Tumour Biol. 2015;36(10):7717‐7723. 25935537 10.1007/s13277-015-3485-0

[45] Yamaguchi M , Suzuki R , Oguchi M . Advances in the treatment of extranodal NK/T‐cell lymphoma, nasal type. Blood. 2018;131(23):2528‐2540. 29602763 10.1182/blood-2017-12-791418

[46] Yamaguchi M , Kwong Y‐L , Kim WS , et al. Phase II study of SMILE chemotherapy for newly diagnosed stage IV, relapsed, or refractory extranodal natural killer (NK)/T‐cell lymphoma, nasal type: the NK‐Cell Tumor Study Group study. J Clin Oncol. 2011;29(33):4410‐4416. 21990393 10.1200/JCO.2011.35.6287

[47] Yamaguchi M , Suzuki R , Oguchi M , et al. Treatments and outcomes of patients with extranodal natural killer/T‐cell lymphoma diagnosed between 2000 and 2013: a cooperative study in Japan. J Clin Oncol. 2017;35(1):32‐39. 28034070 10.1200/JCO.2016.68.1619

