# Supplementary figures and images for: Comparative analysis of clinicopathologic characteristics and prognosis between nasal and nonnasal extranodal NK/T‐cell lymphoma
Source: Cancer Med. 2023 Oct 30;12(23):21138–47. doi: 10.1002/cam4.6674 (PMC10726883; doi:10.1002/cam4.6674)

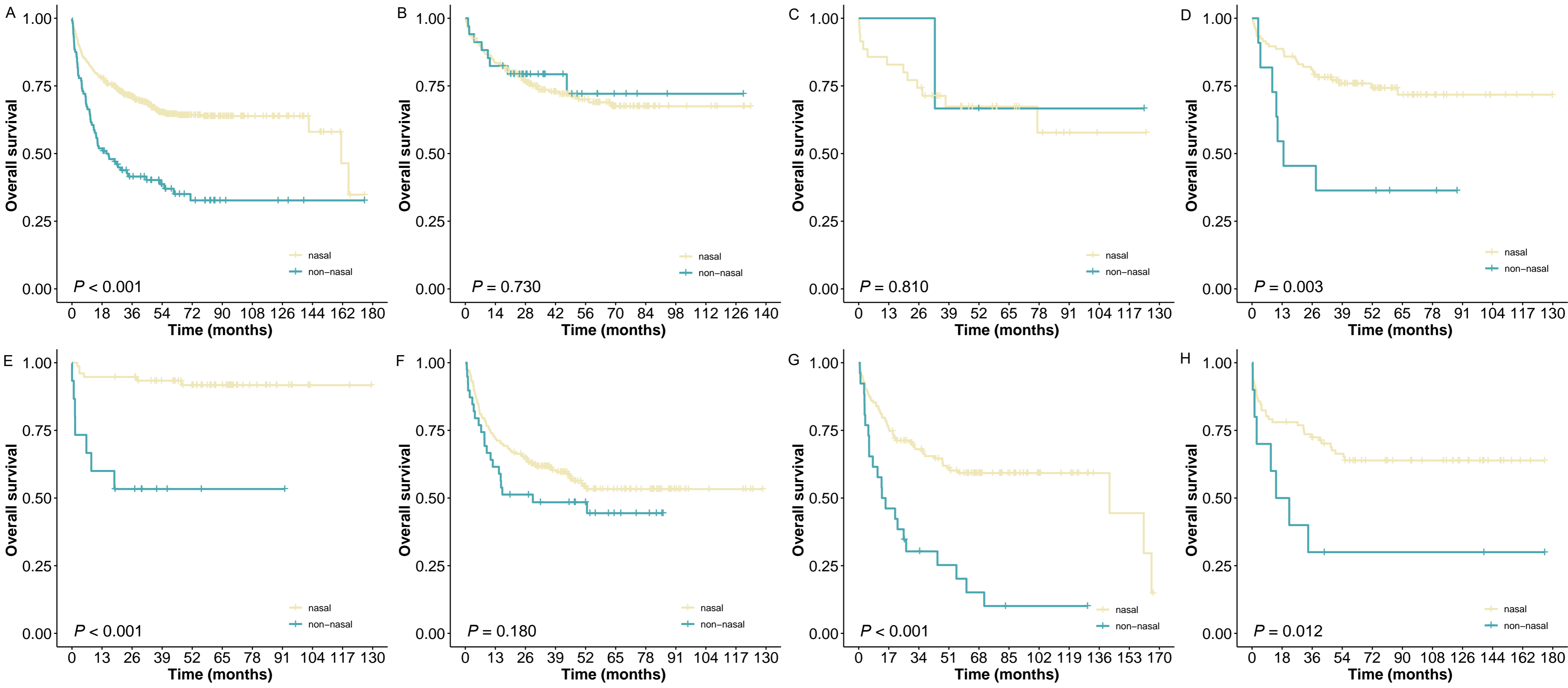

Supplement: Supplementary file 1 — Figure S1. [file CAM4-12-21138-s001.pdf]
